# Molecular Mechanism of Evolution and Human Infection with SARS-CoV-2

**DOI:** 10.3390/v12040428

**Published:** 2020-04-10

**Authors:** Jiahua He, Huanyu Tao, Yumeng Yan, Sheng-You Huang, Yi Xiao

**Affiliations:** Institute of Biophysics, School of Physics, Huazhong University of Science and Technology, Wuhan 430074, China; d201880053@hust.edu.cn (J.H.); d201980099@hust.edu.cn (H.T.); yanyumeng110@gmail.com (Y.Y.)

**Keywords:** coronaviruses, SARS-CoV-2, SARS-CoV, human infection, molecular mechanism, protein docking, MD simulations

## Abstract

The outbreak of a novel coronavirus, which was later formally named the severe acute respiratory coronavirus 2 (SARS-CoV-2), has caused a worldwide public health crisis. Previous studies showed that SARS-CoV-2 is highly homologous to SARS-CoV and infects humans through the binding of the spike protein to ACE2. Here, we have systematically studied the molecular mechanisms of human infection with SARS-CoV-2 and SARS-CoV by protein-protein docking and MD simulations. It was found that SARS-CoV-2 binds ACE2 with a higher affinity than SARS-CoV, which may partly explain that SARS-CoV-2 is much more infectious than SARS-CoV. In addition, the spike protein of SARS-CoV-2 has a significantly lower free energy than that of SARS-CoV, suggesting that SARS-CoV-2 is more stable and may survive a higher temperature than SARS-CoV. This provides insights into the evolution of SARS-CoV-2 because SARS-like coronaviruses have originated in bats. Our computation also suggested that the RBD-ACE2 binding for SARS-CoV-2 is much more temperature-sensitive than that for SARS-CoV. Thus, it is expected that SARS-CoV-2 would decrease its infection ability much faster than SARS-CoV when the temperature rises. These findings would be beneficial for the disease prevention and drug/vaccine development of SARS-CoV-2.

## 1. Introduction

Coronaviruses (CoVs) are a group of enveloped positive-stranded RNA viruses that can cause respiratory, intestinal, and central nervous system infections in humans and animals [1]. Until 2019, six strains of coronaviruses that are able to infect humans had been identified [1,2]. Among them, four human coronaviruses, including HCoV-OC43, HCoV-229E, HCoV-NL63, and HCoVHKU1, are not highly pathogenic and only cause mild respiratory diseases [1]. However, two other coronaviruses, the severe acute respiratory syndrome coronavirus (SARS-CoV) [3,4,5,6] and the Middle East respiratory syndrome coronavirus (MERS-CoV) [7,8], have caused two large-scale pandemics and resulted in more than 8000 cases, including nearly 800 related deaths and about 2500 cases, including about 860 related deaths, respectively. The outbreaks of SARS-CoV and MERS-CoV indicated that some coronaviruses can be highly pathogenic when they transmit from animals to humans [9,10,11]. Therefore, it is urgent to develop antiviral treatments or vaccines targeting such high-risk coronaviruses like SARS-CoV and MERS-CoV.

Before efficient antiviral drugs or vaccines are developed for SARS-CoV or MERS-CoV, another outbreak of pneumonia (COVID-19) caused by a novel coronavirus (SARS-CoV-2) has emerged in Wuhan since December 2019 [12,13,14,15,16,17,18,19]. As of 30 March 2020, SARS-CoV-2 has caused 693,224 confirmed cases, including 33,106 related deaths globally [20]. The full-length genome sequence of SARS-CoV-2 was soon determined by the Zhang group [21]. It was revealed that SARS-CoV-2 has a probable bat origin and is 96% identical at the whole-genome level to a bat SARS-like coronavirus [22]. In addition, SARS-CoV-2 is also closely related to other SARS-like coronaviruses and shares a 79.5% sequence identity to SARS-CoV [22]. For some encoded proteins like coronavirus main proteinase (3CLpro), papain-like protease (PLpro), and RNA-dependent RNA polymerase (RdRp), the sequence identity is even higher and can be as high as 96% between SARS-CoV-2 and SARS-CoV [23]. Therefore, it was thought that SARS-CoV-2 would function in a similar way to SARS-CoV in the human-infection and pathogenic mechanism [22,23,24].

Coronaviruses use the surface spike (S) glycoprotein on the coronavirus envelope to attach host cells and mediate host cell membrane and viral membrane fusion during infection [25]. The spike protein includes two regions, S1 and S2, where S1 is for host cell receptor binding and S2 is for membrane fusion. The S1 region also includes an N-terminal domain (NTD) and three C-terminal domains (CTD1, CTD2, and CTD3) [26]. For SARS-CoV, the receptor binding domain (RBD) is located in the CTD1 of the S1 region. SARS-CoV attaches the human host cells through the binding of the RBD protein to the angiotensin-converting enzyme II (ACE2) [27,28]. Therefore, the interaction between RBD and ACE2 is a prerequisite for the human infection with SARS-CoV. Given the high homology between SARS-CoV and SARS-CoV-2, it was expected that SARS-CoV-2 would also use the ACE2 molecule as the receptor to enter human cells [24,29]. This hypothesis was further experimentally confirmed by the virus infectivity studies from the Shi group, in which SARS-CoV-2 is able to use the ACE2 proteins from humans, Chinese horseshoe bats, and civet as an entry receptor in the ACE2-expressing cells, but not cells without ACE2 [22]. Xu et al. have used MOE to calculate the binding free energies between the Spike-RBD protein and human ACE2, showing that the binding free energy between SARS-CoV-2 RBD and human ACE2 was −50.6 kcal/mol, whereas that between SARS-CoV RBD and human ACE2 was −78.6 kcal/mol [24]. Very recently, the Cryo-EM structure of the SARS-CoV-2 spike protein in the prefusion conformation has been determined [30]. The biophysical and structural evidence suggested that SARS-CoV-2 may bind ACE2 with a much higher affinity than SARS-CoV [30].

Although it has been clear that SARS-CoV-2 infects human cells through the binding of the RBD domain to the human ACE2 receptor [30,31,32,33,34], the molecular mechanism of the binding between the RBD protein and the ACE2 receptor is still unknown. Many questions remain to be answered. For example, previous simulations showed that the binding affinity between SARS-CoV-2 and human ACE2 is weaker than that between SARS-CoV and human ACE2 [24]. However, in reality, SARS-CoV-2 has resulted in many more cases and seems to be more infectious than SARS-CoV. In this study, we have extensively investigated the spike protein/human ACE2 protein systems of SARS-CoV-2 and SARS-CoV by using protein-protein docking and molecular dynamics (MD) simulations. Specifically, we have extensively studied the free energies and dynamics of RBD-ACE2 binding, spike protein, and free RBD protein systems. Given that the spike protein is not only a potential drug target but also the virus antigen [35], the present study will be beneficial for the drug design, vaccine development, and disease prevention of SARS-CoV-2.

## 2. Materials and Methods

### 2.1. Structure Preparation

In this study, we have investigated the RBD-ACE2 complex, spike protein, and free RBD systems of SARS-CoV (GenBank ID: NP_828851.1) and SARS-CoV-2 (GenBank ID: MN908947.3). For SARS-CoV, the RBD-ACE2 complex structure was directly downloaded from the Protein Data Bank (PDB entry: 3SCI) [36]. Then, all the water molecules were removed from the complex structure. The free RBD structure was obtained by removing the ACE2 protein from the RBD-ACE2 complex. The structure of the trimeric spike protein of SARS-CoV was also downloaded from the PDB (PDB entry: 6ACD) [26]. For SARS-CoV-2, the three dimensional (3D) RBD structure was modeled based on the RBD structure of SARS-CoV using the MODELLER program [37], where the sequence alignment was performed using the ClustalW program [38,39]. The complex structure between the SARS-CoV-2 RBD protein and human ACE2 was then predicted by our protein-protein docking approach [40,41,42]. The 3D structure of the trimeric spike protein for SARS-CoV-2 was constructed based on the spike protein structure of SARS-CoV (PDB entry: 6ACD) using MODELLER.

### 2.2. Protein-Protein Docking

The complex structure between the SARS-CoV-2 RBD protein and the human ACE2 molecule was predicted using our hybrid protein-protein docking algorithm, HDOCK [40,41,42,43]. Specifically, given the individual structures of the SARS-CoV-2 RBD protein and the human ACE2 molecule, HDOCK globally samples all possible binding modes between the two proteins through a fast Fourier transform (FFT)-based search strategy [42]. Then, all the sampled binding modes were evaluated by our iterative knowledge-based scoring function ITScorePP [44]. Last, the binding modes were ranked according to their binding energy scores, and the top ten binding modes were provided to users. During the docking calculation, all the default parameters were used. Namely, the grid spacing was set to 1.2 Å for 3D translational search, the angle interval was set to 15${}^{\circ }$ for rotational sampling in 3D Euler space, and the binding interface information in the PDB was automatically applied during the template-based modeling of individual structures. A web server version of our HDOCK algorithm can be freely accessed from our web site at http://hdock.phys.hust.edu.cn/ [41].

### 2.3. MD Simulations

The AMBER suite was used for the MD simulations [45]. Before the simulations, the missing residues in the middle of a chain were added using the MODELLER program [37]. During the simulations, the ff14SB force field was selected [46], an explicit solvent model was used, the time step was set to 2 fs, Langevin dynamics were used for temperature control, and the program “pmemd.cuda” was used as the simulation engine, where all the simulations were performed on a GPU compute node [47]. Specifically, for each system, the following four stages of MD simulations were performed before the production simulation: (1) A 1000-step simulation was first run to minimize the solvated protein system with weakly restraints on the backbone atoms; (2) The system was then heated to 300 K by a 25,000-step (i.e., 50 ps) simulation with weakly restraints on the backbone atoms; (3) Next, another 25,000-step (i.e., 50 ps) constant pressure simulation was conducted to equilibrating the density of the system at 300 K; (4) the system was then equilibrated by a 250,000-step (i.e., 500 ps) of constant pressure simulation at 300 K. Finally, two 2,500,000-step production simulations were run to record the trajectories of the system at 300 K, where the coordinates were written out every 5000 steps (i.e., 10 ps), resulting in a total of 10 ns simulation with 1000 recorded trajectories. After the simulations, the “MMPBSA.py” was used to calculate the free energies of the systems through the MM-GBSA model [48], and the “cpptraj” was used to analyze the coordinate trajectories [49]. To check the statistical significance of the difference between two means, a *t*-test was performed to obtain the P value using two average free energies and their standard deviations.

## 3. Results

### 3.1. The RBD-ACE2 Docking

**Figure 1:**
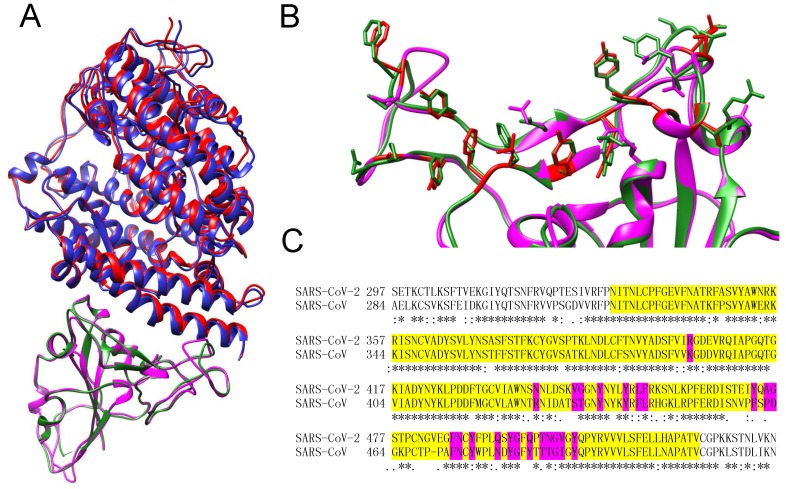
Protein-protein docking with SARS-CoV-2 receptor binding domain (RBD) and human angiotensin-converting enzyme II (ACE2). (**A**) Structural comparison between the predicted SARS-CoV-2 RBD-ACE2 complex and the experimental SARS-CoV RBD-ACE2 structure (PDB code: 3SCI). SARS-CoV-2 RBD is colored magenta and its interacting ACE2 is colored blue. SARS-CoV RBD is colored green and its interacting ACE2 is colored red, respectively. (**B**) The binding site residues on the RBD that are within 5.0 Å form the ACE2, where the hydrophobic residues on the SARS-CoV-2 RBD are highlighted in red. (**C**) Part of the sequence alignment between the spike proteins of SARS-CoV-2 and SARS-CoV, where the RBD residues are highlighted in yellow and the residues at the binding site are highlighted in magenta, respectively.

The RBD proteins of SARS-CoV-2 and SARS-CoV exhibit a high sequence similarity (89.2%) with a sequence identity of 73.7%. The high homology resulted in an accurate 3D RBD model of SARS-CoV-2 with a small RMSD of 0.55 Å from the experimental SARS-CoV RBD structure. With the SARS-CoV-2 RBD model and experimental human ACE2 structure, we then performed protein-protein docking to predict their binding mode using our HDOCK approach [40,41,42]. Figure 1A shows the predicted complex structure between the human ACE2 molecule and the SARS-CoV-2 RBD protein. It can be seen from the figure that the predicted SARS-CoV-2 RBD-ACE2 complex structure is very close to the experimentally determined SARS-CoV RBD-ACE2 complex structure. The interface root mean square deviation (RMSD) between the two complexes is only 0.473 Å, demonstrating that the RBDs of SARS-CoV-2 and SARS-CoV bind to the same site of the human ACE2 receptor (Figure 1A). These results can also be understood by comparing the residues at the RBD-ACE2 binding interface for SARS-CoV-2 and SARS-CoV. The binding sites on the RBD proteins of SARS-CoV-2 and SARS-CoV are very conserved. The corresponding residues show a high sequence similarity of 83.3% (Figure 1B,C). Among them, the hydrophobic residues, which are important for protein-protein interactions, are especially conserved. For the RBD of SARS-CoV-2, there are 13 hydrophobic residues at the binding site, which are comparable to 13 hydrophobic residues for that of SARS-CoV (Figure 1B).

**Figure 2:**
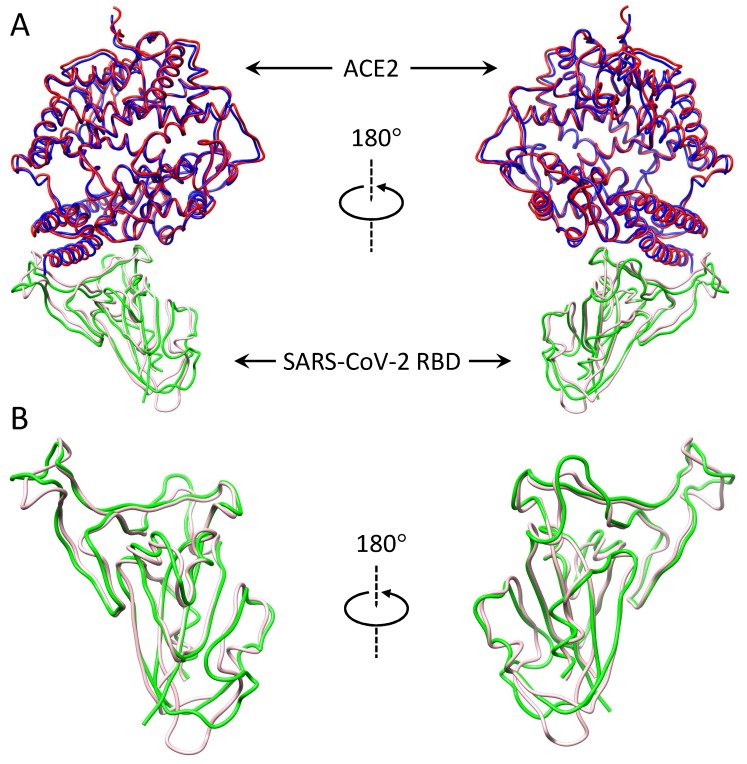
Structural comparison between the predicted and experimental SARS-CoV-2 RBD-ACE2 complexes. (**A**) Alignment of the predicted and experimental SARS-CoV-2 RBD-ACE2 complexes. (**B**) Alignment of the predicted and experimental RBD structures. The RBD and ACE2 of the predicted complex are colored pink and blue. The RBD and ACE2 of the experimental complex (PDB code: 6M17) are colored green and red.

Recently, the CryoEM structure of the SARS-CoV-2 RBD-ACE2 complex was determined by the Zhou group [31]. Comparing the experimental structure and our predicted model showed that the two complexes are close to each other and have a small interface RMSD of 1.108 Å (Figure 2A). The interface difference is mainly due to the conformational changes in the RBD, especially around the loop of residues 475–488 (Figure 2B), while the backbone RMSD for the two ACE2 monomers is only 0.965 Å. If the flexible loop of residues 475–488 in the RBD was excluded, the interface RMSD between the predicted and experimental complexes would be as small as 0.774 Å. These results suggested the accuracy of our predicted SARS-CoV-2 RBD-ACE2 complex structure.

### 3.2. The RBD-ACE2 Interaction: SARS-CoV-2 Binds ACE2 with Higher Affinity than SARS-CoV

We have run a long-time MD simulation to generate the trajectories of the RBD-ACE2 complex system for SARS-CoV-2 and SARS-CoV. The binding free energies were calculated using the MM-GBSA model by the “MMPBSA.py” script in the AMBER package. Table 1 shows a comparison of the RBD-ACE2 binding free energies for SARS-CoV-2 and SARS-CoV. It can be seen from the table that the binding free energy of the SARS-CoV-2 RBD-ACE2 interaction is −50.43 kcal/mol, which is significantly lower than the binding free energy of the SARS-CoV RBD-ACE2 interaction (−36.75 kcal/mol). In other words, SARS-CoV-2 binds human ACE2 with a significantly higher affinity than SARS-CoV. Very recently, an experiment study also suggested that SARS-CoV-2 could bind human ACE2 with a higher affinity than SARS-CoV [30]. This may provide one of the possible reasons why SARS-CoV-2 is much more infectious than SARS-CoV, though other factors like human activities and pathogen persistence can also have a critical impact on the spread of SARS-CoV-2. Further examination of the binding free energy contributions reveals that the higher binding affinity of SARS-CoV-2 than SARS-CoV is mostly attributed to the solvation energy contribution $\Delta G_{\mathrm{solv}}$ (674.97 vs. 696.56 kcal/mol), whereas SARS-CoV-2 has a higher binding free energy in vacuum $\Delta G_{\mathrm{gas}}$ than SARS-CoV (−725.41 vs. −733.31). In other words, SARS-CoV-2 tends to bind human ACE2 better than SARS-CoV in the water, while SARS-CoV would bind to human ACE2 better than SARS-CoV-2 in the gas. Further investigation will be needed to reveal the impact of such binding preferences on human infection.

### 3.3. The Spike Protein: SARS-CoV-2 Is More Stable than SARS-CoV

The spike protein on the coronavirus envelope is a trimeric protein. This protein is critical for the vitality of coronaviruses because it is not only an important component for the virus particle but also plays a crucial role in attaching host cells and fusing the membranes [26]. In addition, the spike protein also determines the solubility of coronavirus particles and thus the viral infectivity because the spike protein is the largest protein located on the coronavirus envelope surface. Therefore, the spike protein is directly related to the stability and functionality of coronaviruses. Here, we have run a lengthy MD simulation to study the trimeric spike proteins of SARS-CoV-2 and SARS-CoV.

Table 2 gives a comparison between the free energies of the spike proteins for SARS-CoV-2 and SARS-CoV. It can be seen from the table that the spike protein of SARS-CoV-2 has a significantly lower total free energy ($G_{\mathrm{total}}=-$67,303.28 kcal/mol) than the spike protein of SARS-CoV ($G_{\mathrm{total}}=-$63,139.96 kcal/mol) (Table 2). These results suggest that SARS-CoV-2 is more stable and able to survive a significantly higher temperature than SARS-CoV. This may partly explain the higher infection ability of SARS-CoV-2 than SARS-CoV because SARS-CoV-2 would have a higher persistence than SARS-CoV at the same temperature.

The lower free energy of the SARS-CoV-2 spike protein may result from the virus evolution or adaption to hosts because SARS-like coronaviruses normally originate from bats that are known to have a higher body temperature than humans [50,51,52]. In other words, SARS-CoV-2 and other SARS-like coronaviruses would have evolved to achieve a lower free energy for their spike proteins by recombination or mutations so that they can survive in high-temperature animals like bats [1]. In addition, the free energy decomposition also shows that the lower free energy of SARS-CoV-2 spike protein than SARS-CoV spike protein is mainly attributed to the free energy in a vacuum $G_{\mathrm{gas}}$ (−36,405.44 vs. −32,053.43 kcal/mol), whereas their solvation energies $G_{\mathrm{solv}}$ are more comparable (−30,897.84 vs. −31,086.53 kcal/mol) (Table 2). This may reflect an evolution trend of SARS-like coronaviruses, i.e., favoring the internal interactions between residues instead of the solvation energy. This kind of evolution would also be beneficial for the persistence of coronaviruses because such kinds of coronaviruses with lower internal energy would be more robust and able to survive in both the air and solvent.

### 3.4. The Free RBD: SARS-CoV-2 Is More Temperature-Sensitive than SARS-CoV

Coronaviruses use the spike protein to attach host cells by binding the host cell receptor. Therefore, the receptor binding domain (RBD) of the spike protein is critical for coronaviruses to infect host cells. Here, we have run lengthy MD simulations to investigate the dynamic properties of the RBD proteins for SARS-CoV-2 and SARS-CoV. Table 3 shows a comparison between the free energies of the free RBD proteins for SARS-CoV-2 and SARS-CoV. Similar to the findings in the spike protein as detailed in the last section (Table 2), the RBD protein of SARS-CoV-2 also shows a significantly lower free energy than that of SARS-CoV (−4090.04 vs. −3617.73 kcal/mol) (Table 3), which may also be understood by the evolution pressure on coronaviruses due to their high-temperature host environment. However, unlike in the spike protein where the free energy difference between SARS-CoV-2 and SARS-CoV is mostly attributed the inter-residue interactions in a vacuum ($G_{\mathrm{gas}}$), here in the RBD protein, the free energy difference between SARS-CoV-2 and SARS-CoV comes from both the free energies in vacuum $G_{\mathrm{gas}}$ (−2104.37 vs. −1703.66 kcal/mol) and solvation energy $G_{\mathrm{solv}}$ (−1985.68 vs. −1914.07 kcal/mol) (Table 3). The lower solvation energy of SARS-CoV-2 RBD than SARS-CoV RBD may be understood because the RBD must move up away from the spike protein and into the water in order to bind human ACE2 [27]. In other words, SARS-CoV-2 would have evolved to be more soluble so that it can move up and bind the ACE2 more easily. The better solubility of SARS-CoV-2 RBD than SARS-CoV RBD may also contribute to part of the higher infection ability of SARS-CoV-2 than SARS-CoV.

**Figure 3:**
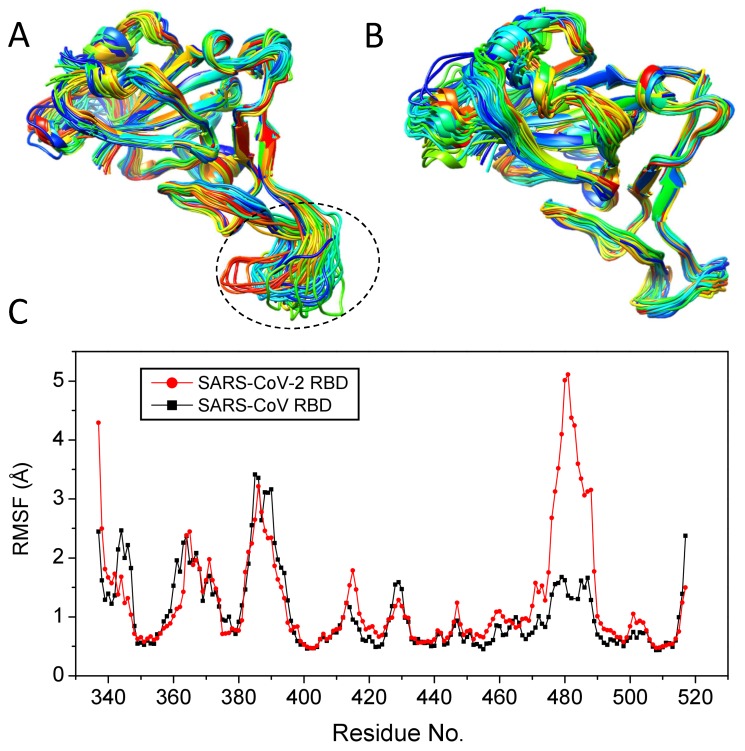
The conformational flexibility of the RBD protein. (**A**) The 50 representative trajectories of the SARS-CoV-2 RBD protein over a 10 ns MD simulation, where the highly flexible region (residues 470–490) is indicated by a circle. (**B**) The 50 representative trajectories of the SARS-CoV RBD protein over a 10 ns MD simulation. (**C**) The RMSFs for the RBD proteins of SARS-CoV-2 and SARS-CoV, where the residue numbering is taken from the SARS-CoV-2 RBD and the data of SARS-CoV are then aligned to those of SARS-CoV-2 according to the sequence alignment.

Protein flexibility is a critical factor in binding as it may not only change the binding interface between two proteins but also be an important contribution to the entropy penalty upon binding. Therefore, we have investigated the protein flexibility of the RBD domains for SARS-CoV-2 and SARS-CoV by analyzing their coordinate trajectories. Figure 3 shows two ensembles of selected trajectories over a period of 10 ns simulations for the RBD proteins of SARS-CoV-2 and SARS-CoV. The figure also gives a comparison of the root mean square fluctuations (RMSF), a rough measurement of protein flexibility, for the RBDs of SARS-CoV-2 and SARS-CoV. It can be seen from the figure that the RBD of SARS-CoV-2 shows a significantly higher RMSF than that of SARS-CoV. In other words, the SARS-CoV-2 RBD is much more flexible than the SARS-CoV RBD. The flexibility is especially higher in the loop of residues 470–490 near the binding site than in other regions (Figure 3A). That means that SARS-CoV-2 must overcome much more entropy penalty than SARS-CoV when binding to human ACE2. As we know, the binding free energy between two proteins can be expressed as, ΔG=ΔE−TΔS, where ΔE is the interaction energy, ΔS is the entropy loss upon binding, and *T* is the temperature of the system. As ΔS is negative here, the binding free energy will become higher and the binding will become weaker with the increasing temperature. Therefore, the RBD-ACE2 binding affinity for SARS-CoV-2 is expected to decrease much faster than that for SARS-CoV when the temperature increases. In other words, SARS-CoV-2 is much more temperature-sensitive than SARS-CoV in terms of RBD-ACE2 binding. Namely, SARS-CoV-2 will decrease its infection ability much faster than SARS-CoV when the temperature rises. Therefore, it is expected that SARS-CoV-2 would become less infectious compared to SARS-CoV, and the disease prevention and control for SARS-CoV-2 may get easier when the weather gets warmer/hotter, although the drug and vaccine development targeting the RBD protein may be more challenging because of the high protein flexibility near the binding site.

## 4. Conclusions

Previous studies showed that SARS-CoV-2 is highly homologous to human SARS-CoV and attaches host cells through the binding of the spike protein to the angiotensin-converting enzyme II (ACE2). However, the molecular mechanisms of SARS-CoV-2 binding to human ACE2 and evolution of SARS-CoV-2 remain unclear. In this study, we have extensively studied the RBD-ACE2 complex, spike protein, and free RBD protein systems of SARS-CoV-2 and SARS-CoV through protein-protein docking and MD simulations. It was found that SARS-CoV-2 can bind human ACE2 with a higher binding affinity than SARS-CoV, which may partly explain that SARS-CoV-2 is much more infectious than SARS-CoV. The spike protein of SARS-CoV-2 shows a lower free energy than that of SARS-CoV, suggesting that SARS-CoV-2 may be more stable and able to survive a higher temperature than SARS-CoV. This may also provide insights into the bat origin of SARS-CoV-2, as bats have a higher body-temperature than humans. In addition, the SARS-CoV-2 RBD exhibits a significantly higher flexibility than SARS-CoV RBD, especially near the binding site. That means that SARS-CoV-2 must overcome a higher entropy penalty in order to bind ACE2 and is thus more temperature-sensitive than SARS-CoV in human infection. Therefore, with the rising temperature, SARS-CoV-2 is expected to decrease its infection ability much faster and become much less infectious than SARS-CoV, which would make the disease prevention and control of SARS-CoV-2 easier. From the above results together, one may infer that unlike SARS-CoV, which was gone after 2003, SARS-CoV-2 might survive a high-temperature environment like Summer in which the virus is not active/infectious due to the high flexibility in the RBD, and then become infectious again when the temperature is low in the Winter. These results may have a far-reaching implication for the disease prevention and control as well as drug and vaccine development for SARS-CoV-2. 

## Figures and Tables

**Table 1:** The binding free energies calculated from molecular dynamics (MD) simulations for the RBD-ACE2 interactions of SARS-CoV-2 and SARS-CoV, where $\Delta G_{\mathrm{gas}}$ is the interaction energy change in the gas and $\Delta G_{\mathrm{solv}}$ is the solvation energy change upon binding in the solvent, respectively. All units are reported in kcal/mol. The *t*-test was conducted to check the statistical significance of the difference between two sets of free energies. A *p*-value of <0.05 indicates that the difference is statistically significant (95% confidence interval).

	SARS-CoV-2		SARS-CoV		*T*-Test	
	Average	Std. Dev.	Average	Std. Dev.	Difference	*p*-Value
$\Delta G_{\mathrm{gas}}$	−725.4066	31.2987	−733.3113	33.8176	7.9047	0.0001
$\Delta G_{\mathrm{solv}}$	674.9721	29.5988	696.5572	29.3955	−21.5851	<0.0001
$\Delta G_{\mathrm{total}}$	−50.4345	5.5811	−36.7541	7.4944	−13.6804	<0.0001

**Table 2:** The free energies calculated from MD simulations for the spike proteins of SARS-CoV-2 and SARS-CoV, where $G_{\mathrm{gas}}$ is the interaction energy in the gas and $G_{\mathrm{solv}}$ is the solvation energy in the solvent, respectively. All units are reported in kcal/mol. The *t*-test was conducted to check the statistical significance of the difference between two sets of free energies. A *p*-value of <0.05 indicates that the difference is statistically significant (95% confidence interval).

	SARS-CoV-2		SARS-CoV		*T*-Test	
	Average	Std. Dev.	Average	Std. Dev.	Difference	*p*-Value
$G_{\mathrm{gas}}$	−36,405.4424	294.4540	−32,053.4298	270.8693	−4352.0126	<0.0001
$G_{\mathrm{solv}}$	−30,897.8370	283.0603	−31,086.5339	236.9148	188.6969	<0.0001
$G_{\mathrm{total}}$	−67,303.2794	171.9868	−63,139.9637	199.4728	−4163.3157	<0.0001

**Table 3:** The free energies calculated from MD simulations for the RBD proteins of SARS-CoV-2 and SARS-CoV, where $G_{\mathrm{gas}}$ is the interaction energy in the gas and $G_{\mathrm{solv}}$ is the solvation energy in the solvent, respectively. All units are reported in kcal/mol. The *t*-test was conducted to check the statistical significance of the difference between two sets of free energies. A *p*-value of <0.05 indicates that the difference is statistically significant (95% confidence interval).

	SARS-CoV-2		SARS-CoV		*T*-Test	
	Average	Std. Dev.	Average	Std. Dev.	Difference	*p*-Value
$G_{\mathrm{gas}}$	−2104.3687	72.3143	−1703.6586	75.9595	−400.7101	<0.0001
$G_{\mathrm{solv}}$	−1985.6759	51.4409	−1914.0726	56.7862	−71.6033	<0.0001
$G_{\mathrm{total}}$	−4090.0446	40.6600	−3617.7312	38.9307	−472.3134	<0.0001
